# Glycolytic Response to Inflammation Over Time: Role of Myeloid HIF-1alpha

**DOI:** 10.3389/fphys.2018.01624

**Published:** 2018-11-22

**Authors:** Susan F. Fitzpatrick, Milos Gojkovic, David Macias, Tetyana Tegnebratt, Li Lu, Erik Samén, Helene Rundqvist, Randall S. Johnson

**Affiliations:** ^1^Department of Physiology, Development and Neuroscience, University of Cambridge, Cambridge, United Kingdom; ^2^Department of Cell and Molecular Biology, Karolinska Institutet, Stockholm, Sweden; ^3^Department of Clinical Neurosciences, Karolinska Institutet, Stockholm, Sweden; ^4^Department of Neuroradiology, Karolinska Experimental Research and Imaging Center, Karolinska University Hospital, Stockholm, Sweden; ^5^Department of Comparative Medicine, Karolinska Experimental Research and Imaging Center, Karolinska University Hospital, Stockholm, Sweden

**Keywords:** HIF-1, myeloid, temporal dynamics, physiological consequences, sepsis

## Abstract

The *in vivo* response to lipopolysaccharide (LPS) occurs rapidly and has profound physiological and metabolic effects. The hypoxia inducible (HIF) transcription factor is an intrinsic and essential part of inflammation, and is induced by LPS. To determine the importance of the HIF response in regulating metabolism following an LPS response, glucose uptake was quantified in a time dependent manner in mice lacking HIF-1α in myeloid cells. We found that deletion of HIF-1α has an acute protective effect on LPS-induced hypoglycemia. Furthermore, reduced glucose uptake was observed in the heart and brown fat, in a time dependent manner, following loss of HIF-1α. To determine the physiological significance of these findings, cardiovascular, body temperature, and blood pressure changes were subsequently quantified in real time using radiotelemetry measurements. These studies reveal the temporal aspects of HIF-1α as a regulator of the metabolic response to acute LPS-induced inflammation.

## Introduction

Hypoxia-inducible transcription factor HIF-1α is one of the key regulators of the cellular adaptation to low oxygen stress. Its expression is controlled both transcriptionally and post-translationally during bacterial sepsis, with previous studies having demonstrated that HIF-1α in myeloid cells plays a key role in regulating proinflammatory gene expression and cytokine production, as well as the mortality and clinical symptomatology of both gram negative (Peyssonnaux et al., 2007) and gram positive (Mahabeleshwar et al., 2011) sepsis.

A growing body of research suggests that myeloid cells affect whole organism metabolism. Recent studies have shown that metabolic adaptations play a key role in myeloid cell response to stimuli and subsequent functional plasticity (reviewed O’Neill and Pearce, 2016). Furthermore, through their expression of different mediators, myeloid cells regulate numerous aspects of peripheral metabolism, including glycolysis and metabolic pathologies such as diabetes (Biswas and Mantovani, 2012). However, the mechanisms through which myeloid cells regulate peripheral glycolysis remain to be fully elucidated. HIF-1α regulates pathways essential for the maintenance of energy homeostasis in myeloid cell types (Cramer et al., 2003). Thus, myeloid HIF-1α may represent an important link between the immune system, peripheral metabolism, and inflammation in sepsis.

The dynamics of the HIF-1α response are complex. Several studies have shown that there is an acute transient accumulation of HIF-1α during hypoxic exposure, both *in vitro* and *in vivo* (Yu et al., 1998; Stroka et al., 2001) and in response to infectious agents (Frede et al., 2006). Thus, we hypothesis that the timing of myeloid HIF-1α accumulation and activation has a significant impact on the transcription of the downstream mediators of the peripheral glycolytic response, as well as on cross-talk with other essential signaling systems during the acute response to sepsis.

To test this hypothesis, we used mice with a targeted deletion of HIF-1α in the myeloid lineage, exposed to lipopolysaccharide (LPS) so as to induce a septic response. We monitored the time-dependent impact of myeloid HIF-1 on the peripheral glycolytic response, organ glucose uptake, body temperature, heart rate, and systolic and diastolic blood pressure.

Computational modeling of the temporal dynamics of the HIF-1α response to hypoxia has indicated that regulation of HIF-1α is can be clearly mapped to the presence of defined negative feedback loops (Cavadas et al., 2013). These include the activation of HIF-1α regulated prolyl hydroxylase (PHD) 2 and 3 enzymes (Leedale et al., 2014), as well as other HIF regulators such as miR155 (Bruning et al., 2011), miR-199a-5p, miR-93, and cRel (Jiang et al., 2015).

Experiments have shown that the timing and magnitude of the HIF-1α response varies based on the cell type and oxygen concentration (Yu et al., 1998). This has been predicted to be due to differences in the ratio of expression of the PHD hydroxylases that regulate HIF post-translationally and expression of HIF subunits themselves. The PHD:HIF synthesis ratio appears relevant where a high ratio causes a sharp transient increase in HIF expression, and in circumstances where a low ratio leads to a delayed response (Qutub and Popel, 2007). The mechanisms controlling the PHD:HIF synthesis ratio are suggested to be due to three feedback loops: autocrine HIF production; an induced expression of the primary PHD in HIF regulation, PHD2; and succinate and inhibition of HIF hydroxylation (Qutub and Popel, 2007). This prediction is supported by *in vitro* work reporting a flexible oxygen threshold for the PHD:HIF response (Stiehl et al., 2006). The work we describe below shows how these various factors impinge on whole animal metabolic response to the stress of sepsis.

## Materials and Methods

### Transgenic Mice

HIF-1 double floxed (DF)/LysM-Cre transgenic mice in C57BL/6 background were generated as previously described (Takeda et al., 2010). Cre-negative homozygous littermates for the conditional alleles were used as controls. Animals were all male and were aged between 8 and 12 weeks at the time of the experiments. All animal experiments were approved by the University of Cambridge Ethics committee and performed according to accepted veterinary standards. Ultrapure LPS (InvivoGen tlrl-3pelps) was administered I.p. (15 mg/kg) for all experiments.

### Glucose Measurements

Blood, isolated at baseline and 1, 2, 4, and 6 h post-LPS injection by tail bleed, was placed onto a glucose StatStrip Xpress (Nova Biomedical, Waltham, MA, United States). Glucose levels were subsequently quantified using a glucometer.

### MicroPET Imaging

Animals were divided into six treatment groups; (18F)-FDG alone; LPS and (18F)-FDG, simultaneous, LPS and 60 min later (18F)-FDG, LPS and 120 min later (18F)-FDG, LPS and 4 h later (18F)-FDG, LPS and 6 h later (18F)-FDG. The (18F)-FDG (obtained as an aliquot from daily clinical productions at Karolinska University Hospital, Solna, Sweden; 7–8 MBq per mouse, maximum volume of 200 μl) was administered to awake, warmed (37°C) mice by a bolus injection via the tail vein. 40 min after the tracer injection, animals were anesthetized with isoflurane (5% initially, 1.5% maintenance) and placed on a heated pad (37°C). Emission data were collected for 20 min in list mode and processed using the MicroPET Focus 120 scanner (CTI Concorde Microsystems). PET data were acquired in 3D mode and images were reconstructed by standard 2-D filtered back projection using a ramp filter. The matrix size of the reconstructed images was 128 × 128 × 95 with a spatial resolution of 1.3 mm.

The 18F-FDG uptake in heart and brown tissues was quantified as standard uptake values, SUVmax (the highest SUV in a region of interest, ROI) and SUVmean (the average intensity of uptake in a ROI), using Inveon Research Workplace software (Siemens Medical Solutions) and normalized to the administered activity (MBq/body weight, gram).

### Oxymax Studies

Energy expenditure was measured using the Oxymax Metabolic Chambers (Columbus Instruments, Columbus, OH, United States). HIF-1DF and HIF-1DF/LysM mice were randomly allocated to the chambers where they had free access top food and water. Baseline VO_2_, VCO_2_, and RER readings were recorded for 48 h but the data for the initial 24 h was discarded. Once mice had acclimatized to the chamber, LPS (15 mg/kg) was administered by I.p. injection and recordings were obtained for a further 6 h.

### Flow Cytometry

Mice aged between 8 to 12 weeks were injected with LPS and kept for a maximum of 2 or 6 h until axillary brown adipose tissue and hearts were collected. Single cell suspensions were prepared using adipose tissue dissociation Kit (Miltenyi Biotec) and heart dissociation protocol (Miltenyi). Once a single cell suspension was obtained, the cells were stained with viability stain (Thermo Fisher) according to manufacturer’s instructions, followed by an FC block (Biolegend). The antibody mix used for staining of different populations were anti mouse CD11b, NK1.1, B220, MHC II, Ly6G, CD11c, Ly6C (BD Bioscience), CD45.2, NK1.1 (Biolegend), for myeloid staining and CD45.2, NK1.1, B220, γδ TCR (BD Bioscience), CD3, CD4, and CD8 (Biolegend) for lymphoid staining. A complete list of fluorochromes, concentrations and clones used for each antibody is shown in Table 1.

**Table 1:** Antibody fluorochrome, concentration, and clones used for flow cytometry experiments.

Stain name	Antigen	Fluorochrome	Dilution 1:x	Clone
Lymphoid	Live/dead	APC-Cy7	500	
Lymphoid	Fc block		50	93
Lymphoid	CD45.2	Brilliant Violet 421	200	104
Lymphoid	CD8	Brilliant Violet 510	400	53-6,7
Lymphoid	B220	Alexa Fluor 488	200	RA3-6B2
Lymphoid	GD TCR	PE	200	GL3
Lymphoid	CD3	PerCP/Cy5.5	100	17A2
Lymphoid	CD4	PE-Cy7	400	GK1.6
Lymphoid	NK1.1	APC	286	PK136
Myeloid	Live/dead	Aqua	500	
Myeloid	Fc block		50	93
Myeloid	CD11b	Horizon V450	200	M1/70
Myeloid	NK1.1	Alexa Fluor 488	200	RA3-6B2
Myeloid	B220	Alexa Fluor 488	200	PK136
Myeloid	Ly6G	PerCP/Cy5.5	500	1A8
Myeloid	CD11c	PE-Cy7	125	HL3
Myeloid	CD45.2	APC	200	104
Myeloid	Ly6C	APC-H7	500	AL-21

### Cytokines Array

Blood was isolated from HIF-1DF and HIF-1DF/LysM mice at baseline and 1, 4, and 6 h post-LPS. Plasma was subsequently isolated and cytokine concentrations were quantified using an automated multiplex assay kit (MSD, Gaithersburg, MD, United States).

### Radio-Telemetry Studies

All radiotelemetry experiments were performed using hardware and software from Data Science International. Radio-transmitters were surgically implanted, under aseptic conditions, into HIF-1DF and HIF-1/LysM mice weighing > 25 g, under anesthesia. 10 days post-surgery the transmitters were turned on and baseline temperature, blood pressure and heart rate recordings were obtained. LPS or saline I.p. was administered under brief anesthesia, due to the presence of the catheter. Following recovery (∼5–10 min) recordings were obtained for a further 6 h. Data acquisition was performed in a designated quiet room to ensure accurate results.

### Statistical Analysis

Statistical differences between groups, was evaluated by Student’s *t*-test or two-way ANOVA. Statistical significance was accepted when *p* < 0.05.

## Results

**FIGURE 1:**
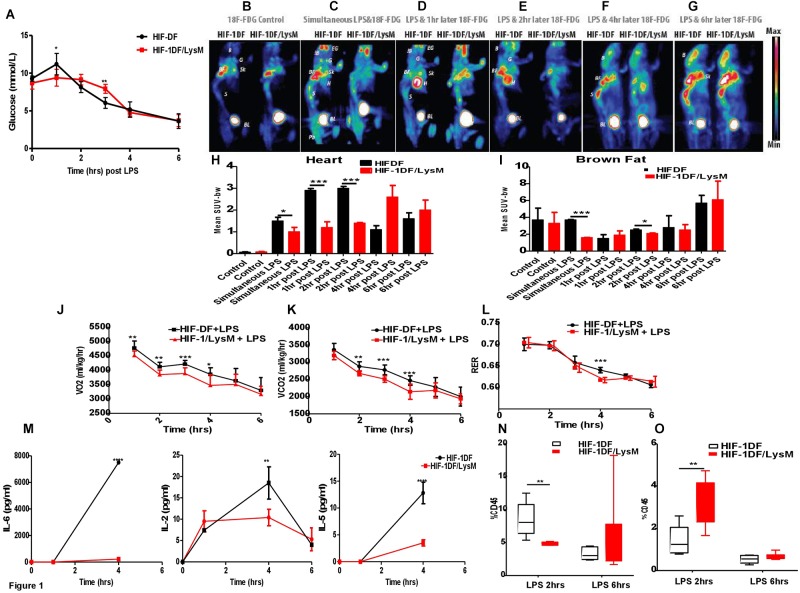
Myeloid HIF-1α is an early regulator of the metabolic and inflammatory response to LPS *in vivo*. HIF-1DF and HIF-1DF/LysM mice were treated with LPS (15 mg/kg). **(A)** Blood Glucose were quantified using a glucometer. **(B)** Mice were given of FDG and control images were obtained. In addition to FDG mice were also given and I.p. injection of LPS (15 mg/kg) **(C)** simultaneously, **(D)** 1 h post, **(E)** 2 h post, **(F)** 4 h post, or **(G)** 6 h post-LPS injection. Animals were anesthetized and imaged for 25 min. Quantification of the of FDG uptake in the **(H)** heart and **(I)** brown fat. Animals were placed in an Oxymax metabolic chamber and **(J)** VO_2_ **(K)** VCO_2_ and **(L)** RER recordings were recorded. **(M)** Blood was isolated from HIF-DF and HIF-1DF/LysM mice at baseline and 1, 4, and 6 h post-LPS I.p. IL-6, IL-5, and IL-2 levels were subsequently quantified. Hearts were isolated from mice 2 and 6 h after LPS treatment and **(N)** CD4 and **(O)** Macrophage infiltration was quantified by flow cytometry. *N* = 4–7. ^∗^*p* < 0.05, ^∗∗^*p* < 0.01, ^∗∗∗^*p* < 0.005, ^∗∗∗∗^*p* < 0.001 versus control (EG-eye periorbital glands; B-whole brain; G-parotid+submandibular glands; BF-brown fat; H-Heart; Sk-scapula, S-spinal cord; BL-bladder; Pb-pelvic).

HIF-1α is an essential regulator of the glycolytic response in macrophages during hypoxia (Cramer et al., 2003). Thus, an initial aspect of our investigation of the temporal role of the factor during inflammation was to assay the role played by myeloid HIF-1a expression on glucose uptake. We observed that when HIF-1DF control littermates were treated with LPS, hyperglycemia occurred after 1 h (see Figure 1). This was subsequently followed by a progressive hypoglycemia from 2 h on after injection. In comparison, loss of myeloid HIF-1α not only protected against the initial hyperglycemia but caused a significant delay in the development of hypoglycemia (Figure 1A).

**FIGURE 2:**
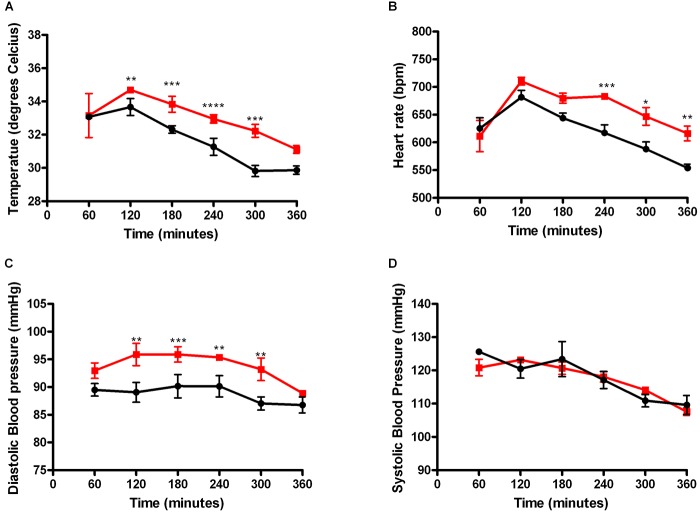
Myeloid HIF-1α plays a time dependent role in regulating clinical symptomology associated with LPS induced sepsis. Real time recordings of **(A)** Temperature, **(B)** Systolic blood pressure, **(C)** Diastolic blood pressure, and **(D)** Heart rate were recorded in mice 10 days following radio-telemetry implantation. *N* = 4. ^∗^*p* < 0.05, ^∗∗^*p* < 0.01, ^∗∗∗^*p* < 0.005, ^∗∗∗∗^*p* < 0.001 versus control.

Sepsis induced hypoglycemia may be due to an enhanced glucose utilization by specific organs, including brown fat, skeletal muscle, or the liver (Brealey and Singer, 2009). To determine this, we used 18F-FDG PET to investigate the time dependent contribution of myeloid HIF-1α to whole organ glucose utilization. 18F-FDG up-take was comparable between the HIF-DF and HIF-1DF/LysM mice at baseline, prior to LPS administration (Figures 1B,I). However, in HIF-1DF controls, 18F-FDG accumulated in the brown fat when administered simultaneously (Figures 1C,I) or 2 h (Figures 1E,I) post-LPS.

In control littermates, 18F-FDG accumulated in the heart at 1 h (Figures 1D,H) and 2 h (Figures 1E,H) after LPS administration. In contrast, this pattern of 18F-FDG up-take was dramatically suppressed in the HIF-1DF/LysM mice lacking HIF-1a in myeloid cells. Interestingly, at those time points (i.e., at 4 and 6 h post-administration of LPS) when blood glucose levels were comparable between the two groups, no differences in whole-organ glucose up-take were observed (Figures 1F–H). This data supports an important role of innate immune cells in determining whole-organ glucose metabolism in LPS-induced sepsis, and identifies HIF-1α as a critical early mediator of this metabolic response.

We next assayed overall metabolic rate via determination of oxygen uptake and carbon dioxide generation during LPS response over time. Baseline metabolic rates were similar in wild type littermate controls and mutants (Supplementary Figure S1). However, HIF-1α myeloid null mice displayed reduced VO_2_ (Figure 1J) between 1 and 4 h and reduced VCO_2_ (Figure 1K) between 2 and 4 h post-LPS administration. Interestingly, while baseline RER measurements were within the normal range of 0.7–0.8 during the inactive light phase, and 0.9–1 during the active dark phase, administration of LPS caused a reduction in respiratory exchange ratios (RERs) values to 0.7–0.65. This implies that the LPS treated mice are using fat as the main source of energy. Such dramatic increases in fat metabolism would be expected in order to try to prevent the severe hypothermia and other symptomatology discussed below. The mutant mice showed lower RER 4 h post-LPS administration (Figure 1L). The lower metabolic rates at earlier time points indicate that a reduced metabolic response by the mutant myeloid cells affects whole animal metabolism. The reduced RER demonstrates a lessening of the glycolytic shift in metabolism in animals lacking HIF-1a in the myeloid lineage. Overall, these results show the essentiality of a HIF-1a response in myeloid cells for inducing a metabolic glycolytic response in this model of septic shock.

HIF-1α also plays a key role in controlling pro-inflammatory signaling pathways. We next investigated the time-dependent role of myeloid HIF-1α in regulating cytokine release in response to LPS. Interestingly, loss of myeloid HIF-1α did not affect LPS-induced release of TNF-alpha, IFN-gamma, IL-12p70, IL-1 beta, IL-10, or KC/GRO (Supplementary Figure S2) at the time points examined. However, in serum isolated from HIF-1DF/LysM mice, lower concentrations of IL-6, IL-2, and IL-5 was observed at 4 h (Figure 1M).

In addition to cytokine release, changes in immune cell activation and infiltration also occur in septic patients. Loss of myeloid HIF-1 caused a reduction in CD4 T cell infiltration (Figure 1N), but increased macrophage infiltration into the heart after 2 h (Figure 1O). By 6 h, levels of immune cell infiltration into the heart were comparable between the HIF-1DF controls and the HIF-1DF/LysM mice (Figures 1N,O). The gating strategy for the myeloid and lymphoid analysis is shown in Supplementary Figures S3, S4 respectively. No significant differences in other immune cell infiltration was observed between the two groups (Supplementary Figure S5).

A striking finding of our 18F-FDG PET study was the accumulation of FDG in the brown fat and heart, which play key roles in thermoregulation and blood pressure respectively. We therefore hypothesized that the accumulation of 18F-FDG in these organs impacts physiological symptomatology in a HIF-1α- and time-dependent manner. To test this hypothesis, temperature, blood pressure and heart rates were recorded in real-time, in HIF-1DF control littermates or in HIF-1DF/LysM mutant mice with radio-telemetry probes.

Baseline analysis between the HIF-DF and the HIF-1DF/LysM mice did not reveal any differences in body temperature, heart rate, systolic or diastolic blood pressure (Supplementary Figure S6). Hypothermia was detected within 3 h of LPS administration in the HIF-1DF wild type control mice. However, the mutant mice were better able to preserve their body temperature at early time points (Figure 2A). While heart rate levels were comparable in the mutant and control animals by 7 h, LPS-induced tachycardia occurred significantly earlier in the control mice in comparison to HIF-1DF/LysM mutant mice (Figure 2B). Similarly, an early protective effect on the diastolic blood pressure was observed in the HIF-1DF/LysM mutant mice (Figure 2C). However, no protective effect was observed on systolic blood pressure measurements (Figure 2D).

## Discussion

HIF-1α plays a critical role in both normal physiology and pathophysiological conditions. In response to hypoxia, infectious and inflammatory agents, there is an acute transient activation of HIF-1α which promotes cell adaptation and survival. However, the dynamics of the HIF-1α response are complex, and the consequences on cell fate and subsequent physiological outcomes *in vivo* remain to be elucidated.

The results of this work extend the observations of previous studies (Peyssonnaux et al., 2007; Mahabeleshwar et al., 2011), which demonstrated that myeloid HIF-1α plays an essential role in the host response to bacterial sepsis. We have shown that myeloid HIF-1 has a subtle yet significant temporal effect on both the glycolytic and inflammatory response to sepsis and subsequent sepsis symptomology *in vivo.*

Dysregulated glycemic control is common during the course of sepsis. Indeed, hypoglycemia has been found to be an independent risk factor for clinically relevant increases in ICU and hospital mortality rates in septic patients (Park et al., 2012). However, the cellular mechanisms underlying the association between hypoglycemia and poor patient prognoses are poorly understood. Our results show that myeloid HIF-1α activation is an acute control element of the glycemic state during sepsis.

One important factor governing plasma glucose concentration are the organ specific aspects of these changes. We observed that the absence of myeloid HIF-1α caused a time-dependent shift in glucose uptake into the heart. Interestingly, a recent study has shown that the administration of 2-deoxyglucose, an inhibitor of the initial kinase (hexokinase-2) for glycolysis, markedly improved cardiac function and survival outcomes in septic mice. The mechanism involved attenuation of the pro-inflammatory response and myocardial apoptosis through suppression of MAP kinase kinase 3 phosphorylation and increased Sirt1 and 3 expression (Zheng et al., 2017).

Consistent with these findings, we observed a correlation between the time-dependent protective effects of loss of myeloid HIF-1α on cardiac glucose uptake and cardiac dysfunction. Autopsy findings suggest immune cell infiltration contributes to that structural changes in the heart and to cardiac dysfunction in septic patients (Smeding et al., 2012). We showed that myeloid HIF-1α plays a key role in regulating immune cell infiltration during the early response to LPS. Moreover, the myeloid HIF-1α response leads to a time-dependent activation of the cytokine system, which also could have a pleiotropic effect on cardiac and brown fat dysfunction. In particular, IL-6 an important mediator during the acute response to sepsis that positively correlates with mortality and severity scores in septic patients (Srisangthong et al., 2013), was significantly suppressed in HIF-1DF/LysM mice. Little is known about the correlation between HIF-1α and IL-6 in sepsis, however, in colitis-associated colorectal cancer serum concentrations of IL-6 positively correlated with HIF-1α mRNA levels in the tumor tissue (Han et al., 2016). Consistent with these findings our results suggest that myeloid HIF-1 plays a vital role in regulating IL-6 in sepsis. Furthermore, our results suggest that myeloid HIF-1α regulated glucose flux plays an important role in cardiac pro-inflammatory response and subsequent cardiac dysfunction.

Hypothermia is another important consequence of sepsis, which has been attributed to increased inflammation and altered glucose utilization (Drosatos et al., 2015). Tissue macrophages maintain sympathetic innervation of the adipose tissue to regulate energy expenditure (Wolf et al., 2017). Heat production requires noradrenaline stimulated lipolysis and thermogenic gene expression, which needs increased glucose uptake (Basse et al., 2017). We found that myeloid HIF-1α temporally regulates glucose uptake into the brown fat in response to LPS, and that this correlates with altered thermoregulation. These results imply an important role for myeloid HIF-1 in regulating body temperature in sepsis.

At present we do not understand the mechanism by which myeloid HIF-1 regulates glycemic control or glucose uptake *in vivo*. However, previous work has shown that loss of endothelial HIF-1 suppressed Glut-1 expression, resulting in decreased glucose uptake into organs, including the heart (Huang et al., 2012). Furthermore, overexpression of Glut-1 in macrophages elevates glucose uptake, which in turn induces a reactive oxygen species-driven proinflammatory response (Freemerman et al., 2014). Therefore, it is plausible that myeloid HIF-1 regulates a glucose uptake which in turn drives a proinflammatory response and sepsis symptomatology.

The clinical implications of these findings are significant. HIF-1α inhibitors have been proposed for the treatment of numerous pathophysiological conditions (Park et al., 2004). However, our results highlight that the role of HIF-1α in disease progression may be time-dependent. Therefore, a greater understanding of the physiological consequences of HIF-1α response will be required in order to determine the usefulness of HIF-1α inhibitors in the clinic.

In summary our results propose that the temporal dynamics of the myeloid HIF-1α response during LPS induced sepsis has significant consequences on both metabolic and inflammatory signaling pathways and clinical symptomatology. Considering the interest in targeting HIF-1α, a better understanding of the effects of its dynamic response could help achieve more focused drug therapy.

## Ethics Statement

This study was carried out in accordance with ethical guidelines of the UK Home Office and the Swedish Animal Research Ethical review board.

## Author Contributions

SF designed, performed and interpreted experiments and wrote the manuscript. MG, DM, LL, TT, ES, and HR performed and interpreted experiments. RJ designed and interpreted experiments, led the project and wrote the manuscript.

## Conflict of Interest Statement

The authors declare that the research was conducted in the absence of any commercial or financial relationships that could be construed as a potential conflict of interest.

## Supplementary Material

FIGURE S1 HIF-DF and HIF-1DF/LysM mice were placed into Oxymax metabolic chambers and baseline VO_2_, VCO_2_, and RER measurements were recorded for 48 h. Lined boxes denote the dark cycle. *N* = 4.

FIGURE S2 Blood was isolated from HIF-DF and HIF-1DF/LysM mice at baseline and 1, 4, and 6 h post-LPS I.p. IFN-gamma, IL-10, IL-12p70, IL-1beta, TNF-alpha, and KC/GRO levels were subsequently quantified using an automated multiplex assay kit. *N* = 6 or 7.

FIGURE S3 Representative FACS plots showing the gating strategy in order to identify frequencies for B Cells (CD45^+^, B220^+^, CD3^-^), different T cells populations (CD3^+^ gdT^+^, CD4^+^, CD8^+^), NK cells (NK1.1^+^ CD3^-^).

FIGURE S4 Representative FACS plots showing gating strategy for Neutrophils (CD45^+^, NK1.1^-^, B220^-^, CD11b^hi^, Ly6G^+^), Monocytes (Ly6G^-^ CD11b^hi^ Ly6C^+^), Eosinophils (SSC-A^hi^ Ly6G^+^) and Macrophages (Cd11b^hi^ Ly6C^+^).

FIGURE S5 Analysis of immune cell infiltration in the heart and brown fat of HIF-DF and HIF-1DF/LysM mice 2 and 6 h post-LPS I.p. was performed by flow cytometry. *N* = 6 or 7.

FIGURE S6 Baseline body temperature, heart rate, and systolic and diastolic blood pressure measurements were analyzed for HIF-DF and HIF-1DF/LysM mice. *N* = 4.

## References

[B1] Basse A. Isidor M. Winther S. Skjoldborg N. Murholm M. Andersen E. (2017). Regulation of glycolysis in brown adipocytes by HIF-1α. *Sci. Rep.* 7:4052. 10.1038/s41598-017-04246-y 28642579 PMC5481455

[B2] Biswas S. Mantovani A. (2012). Orchestration of metabolism by macrophages. *Cell Metab.* 15 432–437. 10.1016/j.cmet.2011.11.013 22482726

[B3] Brealey D. Singer M. (2009). Hyperglycemia in critical illness: a review. *J. Diabetes Sci. Technol.* 3 1250–1260. 10.1177/193229680900300604 20144378 PMC2787024

[B4] Bruning U. Cerone L. Neufeld Z. Fitzpatrick S. Cheong A. Scholz C. (2011). MicroRNA-155 promotes resolution of hypoxia-inducible factor 1ˆ activity during prolonged hypoxia. *Mol. Cell. Biol.* 31 4087–4096. 10.1128/MCB.01276-10 21807897 PMC3187364

[B5] Cavadas M. Nguyen L. Cheong A. (2013). Hypoxia-inducible factor (HIF) network: insights from mathematical models. *Cell Commun. Signal.* 11:42. 10.1186/1478-811X-11-42 23758895 PMC3686674

[B6] Cramer T. Yamanishi Y. Clausen B. Forster I. Pawlinski R. Mackman N. (2003). HIF-1 is essential for myeloid cell-mediated inflammation. *Cell* 112 645–657. 10.1016/S0092-8674(03)00154-5 12628185 PMC4480774

[B7] Drosatos K. Lymperopoulos A. Kennel P. Pollak N. Schulze P. Goldberg I. (2015). Pathophysiology of sepsis-related cardiac dysfunction: driven by inflammation, energy mismanagement, or both? *Curr. Heart Fail. Rep.* 12 130–140. 10.1007/s11897-014-0247-z 25475180 PMC4474734

[B8] Frede S. Stockmann C. Freitag P. Fandrey J. (2006). Bacterial lipopolysaccharide induces HIF-1 activation in human monocytes via p44/42 MAPK and NF-kB. *Biochem. J.* 396 517–527. 10.1042/BJ20051839 16533170 PMC1482811

[B9] Freemerman A. Johnson A. Sacks G. Milner J. Kirk E. Troester M. (2014). Metabolic reprogramming of macrophages. *J. Biol. Chem.* 289 7884–7896. 10.1074/jbc.M113.522037 24492615 PMC3953299

[B10] Han J. Xi Q. Meng Q. Liu J. Zhang Y. Han Y. (2016). Interleukin-6 promotes tumor progression in colitis-associated colorectal cancer through HIF-1α regulation. *Oncol. Lett.* 12 4665–4670. 10.3892/ol.2016.5227 28105173 PMC5228480

[B11] Huang Y. Lei L. Liu D. Jovin I. Russell R. Johnson R. (2012). Normal glucose uptake in the brain and heart requires an endothelial cell-specific HIF-1Â -dependent function. *Proc. Natl. Acad. Sci. U.S.A.* 109 17478–17483. 10.1073/pnas.1209281109 23047702 PMC3491491

[B12] Jiang Y. Zhu Y. Wang X. Gong J. Hu C. Guo B. (2015). Temporal regulation of HIF-1 and NF-kappaB in hypoxic hepatocarcinoma cells. *Oncotarget* 6 9409–9419. 25823824 10.18632/oncotarget.3352 PMC4496226

[B13] Leedale J. Herrmann A. Bagnall J. Fercher A. Papkovsky D. Sée V. (2014). Modeling the dynamics of hypoxia inducible factor-1 within single cells and 3D cell culture systems. *Math. Biosci.* 258 33–43. 10.1016/j.mbs.2014.09.007 25245610

[B14] Mahabeleshwar G. Qureshi M. Takami Y. Sharma N. Lingrel J. Jain M. (2011). A myeloid hypoxia-inducible factor 1alpha -like factor 2 pathway regulates gram-positive endotoxin-mediated sepsis. *J. Biol. Chem.* 287 1448–1457. 10.1074/jbc.M111.312702 22110137 PMC3256857

[B15] O’Neill L. Pearce E. (2016). Immunometabolism governs dendritic cell and macrophage function. *J. Exp. Med.* 213 15–23. 10.1084/jem.20151570 26694970 PMC4710204

[B16] Park J. Chun Y. Kim M. (2004). Hypoxia-inducible factor 1-related diseases and prospective therapeutic tools. *J. Pharmacol. Sci.* 94 221–232. 10.1254/jphs.94.221 15037806

[B17] Park S. Kim D. G. Suh G. Y. Kang J. G. Ju Y. S. Lee Y. J. (2012). Mild hypoglycemia is independently associated with increased risk of mortality in patients with sepsis: a 3-year retrospective observational study. *Crit. Care* 16:R189. 10.1186/cc11674 23062226 PMC3682291

[B18] Peyssonnaux C. Cejudo-Martin P. Doedens A. Zinkernagel A. Johnson R. Nizet V. (2007). Cutting edge: essential role of hypoxia inducible factor-1Â in development of lipopolysaccharide-induced sepsis. *J. Immunol.* 178 7516–7519. 10.4049/jimmunol.178.12.7516 17548584

[B19] Qutub A. Popel A. (2007). Three autocrine feedback loops determine HIF1Î ± expression in chronic hypoxia. *Biochim. Biophys. Acta Mol. Cell Res.* 1773 1511–1525. 10.1016/j.bbamcr.2007.07.004 17720260 PMC2094118

[B20] Smeding L. Plotz F. Groeneveld A. Kneyber M. (2012). Structural changes of the heart during severe sepsis or septic shock. *Shock* 37 449–456. 10.1097/SHK.0b013e31824c3238 22301606

[B21] Srisangthong P. Wongsa A. Kittiworawitkul P. Wattanathum A. (2013). Early IL-6 response in sepsis is correlated with mortality and severity score. *Crit. Care* 17(Suppl. 2):34 10.1186/cc11972

[B22] Stiehl D. Wirthner R. Koditz J. Spielmann P. Camenisch G. Wenger R. (2006). Increased prolyl 4-hydroxylase domain proteins compensate for decreased oxygen levels. *J. Biol. Chem.* 281 23482–23491. 10.1074/jbc.M601719200 16790428

[B23] Stroka D. Burkhardt T. Desbaillets I. Wenger R. Neil D. Bauer C. (2001). HIF-1 is expressed in normoxic tissue and displays an organ-specific regulation under systemic hypoxia. *FASEB J.* 15 2445–2453. 10.1096/fj.01-0125com 11689469

[B24] Takeda N. O’Dea E. Doedens A. Kim J. Weidemann A. Stockmann C. (2010). Differential activation and antagonistic function of HIF-Â isoforms in macrophages are essential for NO homeostasis. *Genes Dev.* 24 491–501. 10.1101/gad.1881410 20194441 PMC2827844

[B25] Wolf Y. Boura-Halfon S. Cortese N. Haimon Z. Sar Shalom H. Kuperman Y. (2017). Brown-adipose-tissue macrophages control tissue innervation and homeostatic energy expenditure. *Nat. Immunol.* 18 665–674. 10.1038/ni.3746 28459435 PMC5438596

[B26] Yu A. Frid M. Shimoda L. Wiener C. Stenmark K. Semenza G. (1998). Temporal, spatial, and oxygen-regulated expression of hypoxia-inducible factor-1 in the lung. *Am. J. Physiol. Lung Cell. Mol. Physiol.* 275 L818–L826. 10.1152/ajplung.1998.275.4.L818 9755115

[B27] Zheng Z. Ma H. Zhang X. Tu F. Wang X. Ha T. (2017). Enhanced glycolytic metabolism contributes to cardiac dysfunction in polymicrobial sepsis. *J. Infect. Dis.* 215 1396–1406. 10.1093/infdis/jix138 28368517 PMC5451607

