# Supplementary figures and images for: Glycolytic Response to Inflammation Over Time: Role of Myeloid HIF-1alpha
Source: Front Physiol. 2018 Nov 22;9:1624. doi: 10.3389/fphys.2018.01624 (PMC6262152; doi:10.3389/fphys.2018.01624)

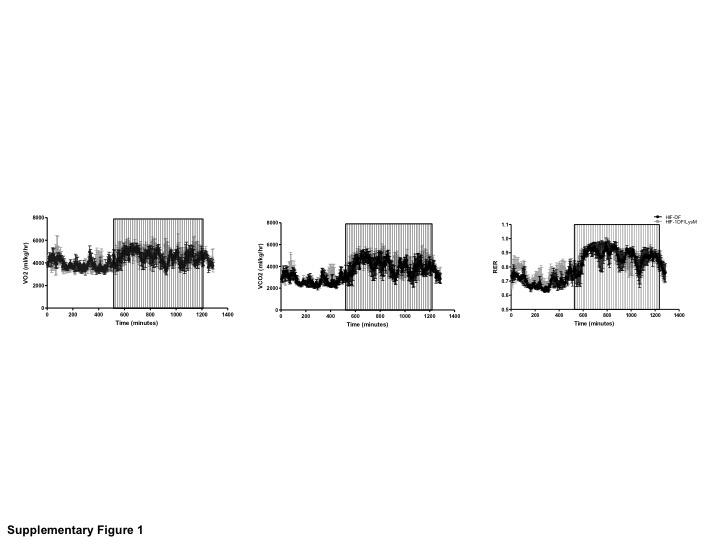

Supplement: FIGURE S1 — HIF-DF and HIF-1DF/LysM mice were placed into Oxymax metabolic chambers and baseline VO2, VCO2, and RER measurements were recorded for 48 h. Lined boxes denote the dark cycle. N = 4. [file Image_1.JPEG]

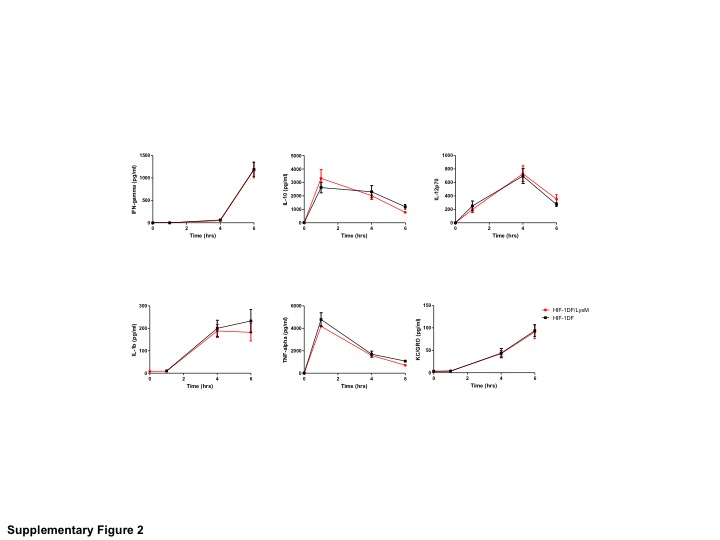

Supplement: FIGURE S2 — Blood was isolated from HIF-DF and HIF-1DF/LysM mice at baseline and 1, 4, and 6 h post-LPS I.p. IFN-gamma, IL-10, IL-12p70, IL-1beta, TNF-alpha, and KC/GRO levels were subsequently quantified using an automated multiplex assay kit. N = 6 or 7. [file Image_2.JPEG]

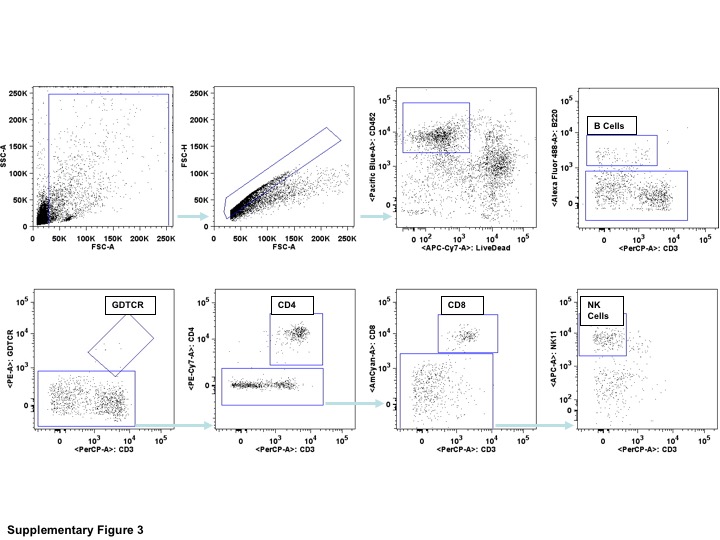

Supplement: FIGURE S3 — Representative FACS plots showing the gating strategy in order to identify frequencies for B Cells (CD45+, B220+, CD3-), different T cells populations (CD3+ gdT+, CD4+, CD8+), NK cells (NK1.1+ CD3-). [file Image_3.JPEG]

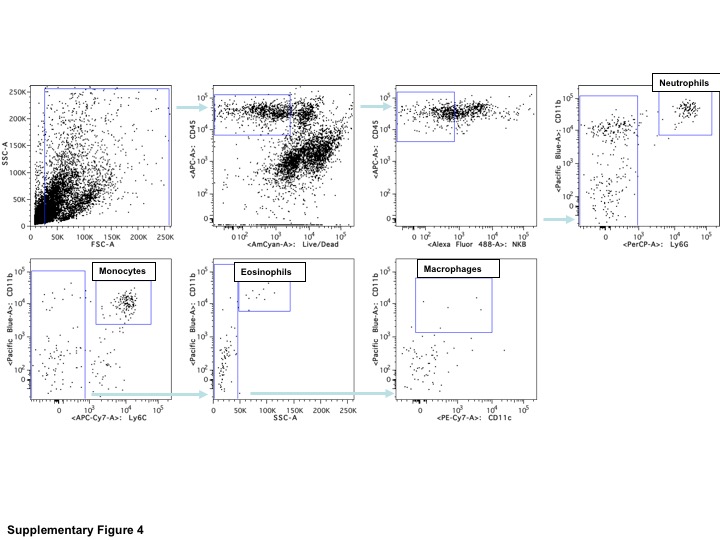

Supplement: FIGURE S4 — Representative FACS plots showing gating strategy for Neutrophils (CD45+, NK1.1-, B220-, CD11bhi, Ly6G+), Monocytes (Ly6G- CD11bhi Ly6C+), Eosinophils (SSC-Ahi Ly6G+) and Macrophages (Cd11bhi Ly6C+). [file Image_4.JPEG]

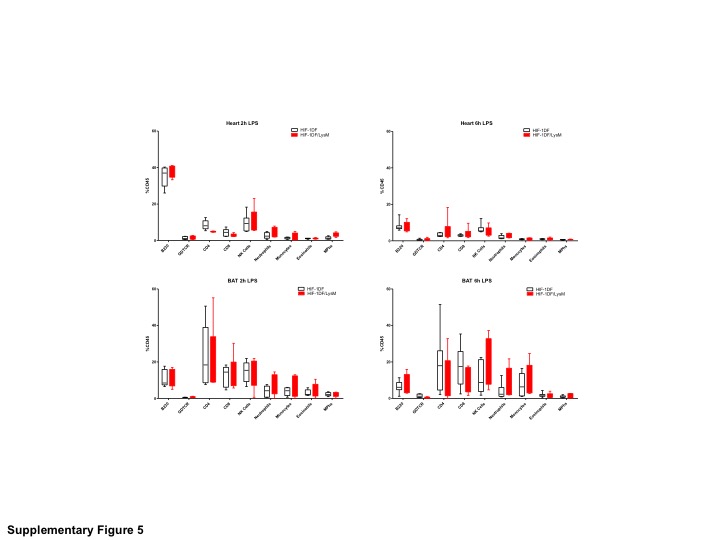

Supplement: FIGURE S5 — Analysis of immune cell infiltration in the heart and brown fat of HIF-DF and HIF-1DF/LysM mice 2 and 6 h post-LPS I.p. was performed by flow cytometry. N = 6 or 7. [file Image_5.JPEG]

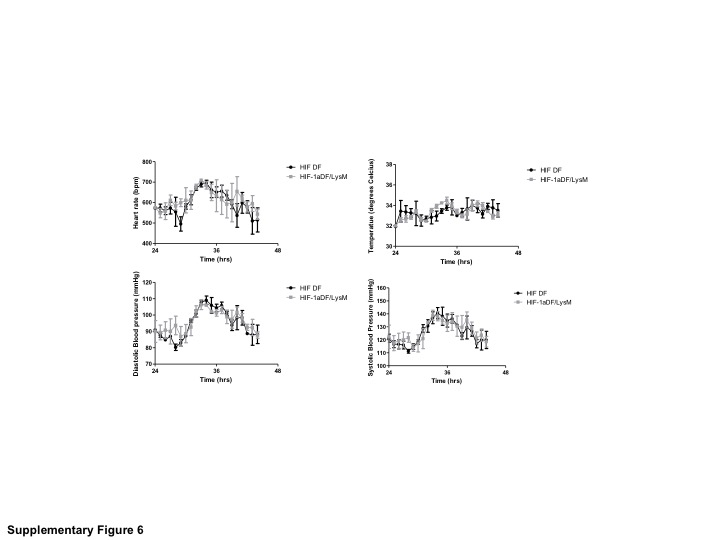

Supplement: FIGURE S6 — Baseline body temperature, heart rate, and systolic and diastolic blood pressure measurements were analyzed for HIF-DF and HIF-1DF/LysM mice. N = 4. [file Image_6.JPEG]
